# On the Prevention of Abscess of the Breast by Frictions with the Hand

**Published:** 1857-10

**Authors:** S. Conant Foster

**Affiliations:** Late Physician to Bellevue Hospital


### SELECTIONS.

*On the Prevention of Abscess of the Breast by Frictions with the hands; and on the Treatment, when op'n, by means of Compressed Sponge.* By S. Conant Foster, M. D., late Physician to Bellevue Hospital.

I have been often asked to commit to paper my views in regard to the treatment of mammary abscess, and now that the method which I have found most beneficial has been tried by others, and has stood the test of their observation, I feel that it has become a duty to publish the results to the profession.

Under the head of "Hospital Reports," will be found a number of cases treated at Bellevue Hospital, under the supervision of Dr. Stephen Smith, by Dr. J. G. Johnson, one of the House Surgeons of that institution. The MSS. of these cases have been kindly submitted to my perusal by Dr. J., to whom I feel much indebted for his courtesy, and for the care which he has bestowed upon them. These cases, in connection with those which I shall offer, afford conclusive evidence, it seems to me, of the superiority of the treatment pursued over the old methods. What these old methods were may be learned from Dr. Johnson's paper, where the authorities will be found collated, and the opinions and advice of the principal surgeons of the present day, quoted in their own words.

Before entering upon the subject of the best mode of treatment of any disease, it is proper to consider if the disease may not be avoided altogether. Now, I do not hesitate to assert, with regard to abscesses of the breast, dependent upon lactation, that this may be done in almost every case. I base this assertion upon the following grounds: 1. During six months that I passed in attendance upon the Dublin Lying-in Hospital, under the mastership of Dr. Evory Kennedy, now nearly twenty years since, there were about twelve hundred births. Only two abscesses of the breast occurred among these patients during their stay in the hospital. One of these was the result of an injury, and came on before confinement; the other was in a patient of marked chachectic condition. 2. For the first twelue years of my practice, I had not a single case occurring to a patient of my own during the first month after delivery; and I have never had one since which was not due to mismanagement on the part of the patient, or want of skill in the nurse, or to an already depraved condition of the blood. 3. For nearly six years that I was visiting physician to Bellevue Hospital, excepting in the first two months, no case occurred among the lying-in patients under my charge, which I attribute to my insisting strenuously upon the adoption of the means I had seen to be so efficacious elsewhere.

Laying it down as a rule, therefore, that *all milk abscesses should be prevented*, the exceptions are—1. Cases where there exists a marked cachexia, due to syphilis, scrofula, and the like. 2. Where there has been any external injury. 3. When the nipple is in such a state as absolutely to obstruct the passage of the milk. 4. Where, as in hospitals occasionally, there exists an endemic tendency to purulent deposits.

Let us now glance at the means by which this prevention is to be accomplished. They will be found to be very simple. So simple, indeed, that I should hesitate to dwell upon them, if I were not convinced that, when they fail, it is for want of a skillful application of them. If, therefore, I should seem to be discoursing of trivialities, I shall shelter myself behind the example of the great Abernethy, who boasted, in his discourse before the College of Surgeons, that he had taught that august body how to make a poultice. I desire, too, to urge upon physicians to devote more personal attention to these apparently little matters; for the most faithful and well-intentioned nurses will often fail, for want of knowledge of the structure and physiology of the part which they have to deal with.

The prophylactic treatment of the milk abscess should commence, strictly speaking, at the age of puberty. This is a matter upon which mothers should be well informed, as more will depend upon them than upon the physician. At the period when the development of the breasts is taking place, much mischief is very often done by the use of improper clothing. A close-fitting waist, keeping the nipple compressed at this period, and preventing it from taking its natural form, though it may occasion no incovenience at the time, will be a fruitful cause of suffering when the girl has become a mother. For, in these cases, it will probably be discovered after confinement, that the nipple, instead of being prominent, is depressed, so that the child cannot seize hold of it. It has then to be drawn out by breast-pumps and other appliances, often at the cost of great pain. It becomes inflamed and excoriated; and, perhaps, the inflammation may be propagated along the course of the lactiferous tubes, and result in an abscess at the base of the breast.

This is a cause of the breast abscess which we seldom have it in our power to prevent. But where a depressed condition of the nipple exists from this early error, much may yet be done if it is permitted to treat it during pregnancy. In this condition, a second development of the organ takes place. There is an increased vascular and nervous activity, by taking advantage of which we may often remedy the old trouble in some degree, and provide a good nipple for the period of lactation. To effect this end, the first precaution is to avoid all undue pressure from clothing, during the earlier months especially. The nipple should then be drawn out daily, with moderate force, by a breast-pump or by the mouth, which accustoms the part to the traction it will receive from the child. This process can be suspended for a few days at a time, if the nipple should become sore. Astringent applications, as is well known, tend to harden the skin of the nipple. Of these I know of none better than the popular one of borax and brandy, which should be applied daily during the last months of gestation.

There is a certain danger attendant upon these manipulations with the nipple during pregnancy, which must not be lost sight of. It is possible, in this way, to stimulate the uterus into premature action, and so to provoke a miscarriage. But this can only occur in exceptional cases, and will probably give sufficient warning of its approach. Lest it should occur, it is well not to persist in drawing out the nipple longer than ten or fifteen minutes at a time, and to abandon the practice altogether should pelvic pains, or other symptoms of approaching labor, manifest themselves. I have never seen a miscarriage so produced; but in one case which I attended, the process had to be given up on account of the severe, contractile, uterine pains which followed each application of the pump.

With the management of the nipple after child-birth, every one is familiar. Probably, under any treatment, we must have sore nipples occasionally. As I have nothing new to offer concerning the treatment of these, I will simply say that I rely more upon the protection of the part from friction by the clothes, upon allowing the air to circulate around it, and upon the application of castor-oil after every time the child sucks, than upon most of the other means in common use. The neck of a common junk bottle, cut off, made smooth, and worn constantly over the nipple, accomplishes the two first of these objects very satisfactorily in most cases.

So much of the prophylaxis of breast abscess as far as the nipple is concerned. As to the general care of the breast after childbirth, the obstetrical text-books give a variety of directions. No doubt the first and most important indication is to prevent the accumulation of milk in the breasts. He who succeeds in doing this has overcome the chief source of danger of abscess. With this in view, I examine the breasts carefully on the second day after delivery, and direct the nurse, in case there should be the slightest hardness, to rub them well with warm oil *until they are soft*, and to repeat the operation as often as the hardness returns. This is a very common and a very simple direction, and yet it is very rarely carried out efficiently. When it is done well, it is worth more than all other means put together to keep the breast in good order. I do not despise cathartics, and nauseants, and suodorfices when inflammation has commenced, though even then I do not rely *chiefly* upon them to prevent the formation of matter; but I must express my disapprobation of poultices with a view to resolve the hardness; and if it were necessary, I would dispense with all other means, excepting the nursing of the child, and rely upon the rubbing alone, always provided it could be done frequently and efficiently.

A great element of success is to commence early—*obsta principiis.* The rubber should stand behind the patient, and having lubricated the whole organ freely with oil, should use both hands, one for the friction and the other for counter-pressure, rubbing in the direction of the milk ducts, *i. e.*, from the periphery of the breast toward the nipple. If this is done with *firm pressure*, a few minutes will generally suffice to reduce the organ to a soft condition; and in case the milk has come, it will flow freely from the nipple. If the child is vigorous and takes the nipple well, there will usually be no need of pursuing this plan after two or three days. But it is desirable to keep an eye upon the organs, and in case any induration remains after nursing, to rub it away at once.

I have intimated that it is difficult to get this well done. This is sometimes the fault of the patient, and sometimes of the nurse. The patient's breasts are very tender to the touch, and she thinks she cannot bear the rubbing. But if it is done rightly, the pain will be of very short duration, and at the end of a few moments she will be able to bear any amount of pressure without suffering in the least. There seems to be a certain amount of tact requisite for this little manipulation, which many nurses never acquire. In such a case, I think it is the duty of the medical attendant to lay aside all considerations of dignity, and do it himself. But as the physician cannot be constantly in attendance, it is necessary, if after one or two trials the induration is not permanently discussed, to resort to other means. And here we have a most invaluable resource in the compressed sponge.

For the suggestion of this mode of treating such engorgements, I am indebted to Dr. J. P. Batchelder, of this city, who has applied this article to so many excellent uses. Its use in the treatment of open breast abscesses I believe I was the first to test, and it is a chief object of this paper to record the results of the method. The mode of preparing the sponges is very simple. A selection should be made of specimens which combine softness with elasticity, and of a size to suit the indurated portion. After freeing them from sand they should be dried, and then subjected to a heavy pressure for twenty-four hours. A common letter-copying press answers the purpose exceedingly well. They are then to be placed upon the breast and confined by a roller bandage, after which they are to be thoroughly saturated with lukewarm water. The nipple should, if possible, be left exposed, so that the child may nurse as usual. I will relate briefly the first case in which I adopted this plan. It has proved equally satisfactory in every instance in which I have employed it since.

A lady whom I had attended in three confinements was, after the third, threatened with abscess in the breast. I directed, as usual, the frictions with warm oil, and administered, subsequently, cathartics, and nauseating doses of tartar emetic. The inflammation was thus kept in check, but the induration continued. This patient had a most faithful nurse, who rubbed the breast very perseveringly for several days, but without much effect. Satisfied that the want of success was due to the manner of the rubbing, I resolved to rub it myself, and in twenty minutes or less, the breast was completely soft. Next day, however, it was nearly as hard as ever. I now determined to comply the compressed sponge. Two days wearing of this entireiy removed the hardness, which did not again recur.

I have pursued the same plan with the like success in engorgements occurring at a later period in lactation, where the frictions with oil had failed to subdue the hardness.

Before proceeding to show the effect of the application of compressed sponges to abscesses which have already formed and been laid open, I desire to call attention to the treatment directed by the most eminent of modern writers on the subject. For this purpose, I beg to refer to the remarks accompanying Dr. Johnson's collection of cases. I also appeal to the experience of every surgeon to bear me out in the assertion that, in the deep-seated abscesses of the breast of nursing women, it is reasonable to anticipate a long and tedious suppuration, with the chance of sinuses and fistulous openings, enduring for many months. Not only is this true, but the plans formerly adopted of stimulating injection, setons, extensive incisions, objectionable as they were, were often unsuccessful, or only cured by destroying the organ in great part, or, at least, rendering it completely unfit for performing its proper function.

The only way of avoiding these evils, that has been attended with any degree of success, is to apply pressure in some form or other, generally by means of strips of adhesive plaster. Recently, M. Chassaignac has published a memoir upon this subject, (*Gazette Medicale,* 1855—Nos. 3, 4, and 39) which details the method and results of a new system of treatment, presenting considerable advantages over all that preceded it.

His plan is as follows:—As soon as the existence of matter is ascertained, he makes one or more small punctures, and having pressed out the matter as much as possible, injects the abscess with lukewarm water, using generally the hydrocele syringe or the irrigator, and continuing the douche until the cavity seems entirely emptied of pus, and the liquid which flows out is perfectly limpid. The cupping glass is then applied over the wound, and, if necessary, the same process is repeated until every drop of pus is evacuated. The wound is then closed by adhesive plaster and the *cuirasse* applied. This consists, if I am not mistaken, of numerous narrow strips of plaster, so adjusted as to envelop and compress the entire breast. The arm is then bound down to the side, and the dressing is allowed to remain twenty-four or forty-eight hours, unless pain or tumefaction indicate the re-filling of the abscess. At the end of this time the dressing is removed, and it is found that union by the first intention has taken place, in the proportion of about one-fifth of all the cases. Sometimes there is a little secretion of plastic lymph, and then the *cuirasse* is re-applied once or twice. A cure is thus accomplished in from one to eight days.

In case the abscess refills, the treatment of union by the second intention consists in the daily, or more frequent use of the injections and cupping, with the addition of the introduction of canulæ into the wounds, made of soft india-rubber, and split at one end, so as to have the shape of the letter Y. These are kept in place by a narrow strip of adhesive plaster passed between the forked ends, and shortened from time to time, as occasion may require. The breast is kept covered with a poultice, and the poultice with oiled silk. In very grave cases, where the abscess is at the very base of the gland, the seton is resorted to.

By these means M. Chassaignac considers, and shows, that the duration of these abscesses is much abbreviated. He says that, since he has adopted them, he never sees the cases last for whole months as was formerly frequent. "A duration of three months, for example, is no longer known in our wards." Of the cases reported, where union took place by the second intention, one was cured in a week, and most of the rest within a month, but it is not pretended that all get well as soon as longest of these periods.

I have been thus particular in furnishing an abstract of M. Chassaignac's method, because I believe it to be little known in this country, while it is eminently worthy of attention. At the same time, I desire to invite a comparison of the results of his treatment and that by the sponges, of which I am now about to relate some cases.

One word as to classification. M. Chassaignac makes two grand divisions, viz.: 1. Extero-mammary. 2. Intry-mammary. The first of these divides into sub-cutaneous and sub-adenoid. The second into interlobular and canalicular. Each of these four is again divided into several varieties, as diffuse, phlegmonous, angioleucitic, etc. While I admire the exceeding nicety in diagnosis of this distinguished surgeon, and have no doubt of the correctness of his classification, yet I submit that, practically, it is not of very material consequence to make such minute distinctions. What we do wish to know, however, in every case is, whether or not the gland itself and the lactiferous tubes are involved. For, in the one case, we *may* allow the child to draw the milk from the breast, while in the other it *must* be strictly forbidden. We know that the tubes are affected, when we see milk mingling with the pus which is discharged from the abscess, or when pus is discharged from the orifices of the nipple. But we cannot always be sure that they are not involved when neither of these things takes place, since the secretion of milk may be entirely arrested, and so not appear at the cut, or pus may be formed at the extremities of the canaliculi, and discharge into the cavity of the abscess, only finding its way through the nipple as the case approximates to a cure. This happened in a very marked manner in one of the cases which I shall relate.

I am unable to furnish exact notes and dates of the three or four cases of *superficial* abscess which I have treated by the sponges. They have, however, all healed up in a manner equally rapid with those reported by Mr. Chassaignac, viz.: in twenty-four to forty-eight hours. In one instance the abscess was on the inner edge of the gland, and of the size of a hen's egg. In another there were two, and on each side, pointing within the areola, occurring on successive days. Only one application of the sponge to each was required, the infant continuing to nurse without interruption. The cases which follow involved a large portion of the organ and the milk tubes.

*Case* 1.—Mrs. H. J. was confined with her first child August 1, 1855. I left town a few days after, and on my return, about the first week in September, found her with a very large abscess, involving nearly the whole breast. The patient, naturally robust, was in a state of extreme prostration. The brerst was opened, in the course of the next few days, in five different places, and discharged an enormous quantity of pus. A probe could be passed its whole length, in different directions, through and under the gland. The entire organ was in a state of induration and enlargement. No milk could be got from the nipple. On September 13, a new point of inflammation having appeared on the upper part, and the skin being already bright red over a space of two inches square, I applied two sponges—one to the newly inflamed part, and the other to the lower part of the breast, covering all the incisions.

September 14.—Inflammation and hardness entirely gone in the first named locality; the size of the breast diminished one-third; the cuts healing, and the milk running freely.

Being unable to obtain a supply of the compressed sponges, I did not repeat them on the 14th; but they were re-applied on the 15th, 17th, and 18th. On this last day, the wounds had all healed, excepting one near the nipple, from which, as well as from the nipple, the milk flowed freely. Breast about one-half the size of the other. Some induration in the lower third only.

September 19.—Further improvement. Sponges omitted—the breast being merely suspended in a sling; patient rode and walked out.

September 20.—Some soreness and induration in upper part of breast. One of the cicatrized points below looking red, I opened it with the end of a probe, and let out two or three drops of pus. From another, which was opened in the same way, a few drops of clear serum flowed. Re-applied sponges.

September 21.—The fistulous opening near the nipple gave exit, after pressure upon the upper part of the breast, to about a drachm and a half of sero-sanguineous matter. The other openings firmly healed again, and the breast was everywhere softer, and free from tenderness.

From this date to the 26th the sponges were applied daily, with one exception. On the 27th, the patient left town on a visit to some friends, the general health entirely restored—the whole breast being soft; the milk flowing very freely; the fistulous opening almost healed, barely receiving the point of the probe, and yielding a single drop of serum. From the outset to the close of this case, there was no pain following the application of the sponges—the patient, on the contrary, obtaining marked relief under their use. A very slight inconvenience was felt, the first day or two, from the tightness of the bandage. The patient was allowed full diet, with wine.

A year afterward, I attended this lady in a second confinement. There was some threatening of inflammation a second time, but it disappeared after one or two rubbings, and she nursed her child on this side as well as the other.

*Case* 2.—Mrs. W. was confined near the last of May, 1856, with her second child. This lady had been attended in her labor by a homœpathist, and was doing well until about the tenth day, (notwithstanding a very constipated state of the bowels, which, as usual, had not been interfered with,) when the breast began to inflame. It was treated lightly by the attendant, who left town, and I was called in on the evening of June 6. Although there was some evidence of suppuration, I yet hoped to avoid an incision, and on the 7th applied the compressed sponges, and continued them until the 10th, with entire relief of the pain, and subsidence of inflammation, when, finding that it was indispensable to evacuate the matter, I ordered a poultice, and opened the abscess on the 11th, giving exit to about half a pint of pus, mixed with milk. On the 12th I applied the sponges again, and continued them daily until the 17th. On the 14th the discharge consisted almost entirely of milky serum.

June 16.—Dressing omitted at night.

June 17.—A soft moist sponge constituted for the compressed.

June 18.—Bandages omitted. Two or three drops of serum only from the cut.

June 20.—Wound perfectly cicatrized; breast free from hardness everywhere, and milk flowing freely.

In this case the sponges were only required for five days after the abscess was opened.

*Case* 3.—Mrs. M., æt. about 20 years, of healthy constitution, was confined on February 25, 1857, with her first child. She had a very tedious labor, in the course of which I was called to see her, in consultation. After delivery she did well for the first week or ten days, when symptoms of breast abscess showed themselves. On March 11, I was again requested to see her, and found her with the right breast very much swollen and hard, yielding no milk; the left also hard, to a less extent, but giving milk; and the patient in a very feeble condition. There was, with all this, very little soreness or acute pain, but the nervous system was so far affected that she could not sleep, and the pulse was upward of one hundred. There was an obscure sense of fluctuation in the right breast, but so uncertain, that I did not feel justified in making an incision. I, therefore, applied a poultice, and, on the next day, finding no change except an increase of tumefaction, and, aggravation of all the symptoms, I decided to apply the sponges. By request of the attending physician, I now took entire charge of the case. The first application was followed by a decided relief of all the symptoms. On the 13th, the matter was apparent enough, and I made an opening, letting out about eight ounces.

On this day, and the 14th, the breast was poulticed, and discharged a large quantity of pus.

March 15.—Sponges applied.

March 16 and 17.—On each of these days new collections of matter were discovered, not communicating with the first, and were opened. After evacuating the matter, which was deep-seated, the sponges were re-applied.

March 18.—After passing out the matter as much as possible, a small slough appeared at the principal wound, which was seized by the forceps and extracted. A second slough now appeared, which was still firmly adherent. The cavity was injected with warm water (which caused no pain), and completely washed out. Poultice.

March 19.—The slough appeared loose, but so large that it was necessary to dilate the wound, after which it was extracted with a dissecting forceps. The removal was followed by the discharge of a large quantity of matter, apparently from the very base of the gland. The slough extracted was as large as a hen's egg. After its removal, the external half of the breast collapsed, forming a considerable depression. Air got into the cavity, and insinuated itself into its ramifications, being perceptible to the touch on the superior and sternal edges, at points farthest removed from the first seat of the matter. Poultice applied this day and the next.

March 21.—The discharge having somewhat diminished, the sponges were re-applied.

March 22.—Same. Discharge very much diminished, and the breast everywhere softer.

March 23.—Further improvement. On gently rubbing the breast with oil, a few drops of milk appeared at the nipple. General condition very much improved. Gaining strength daily.

From this date until April 6, the sponges were applied daily. At each dressing the breast was rubbed with oil. The secretion of milk was re-established—a certain quantity flowing spontaneonsly, and two or three ounces obtained from the nipple at each dressing. Milk also flowed from the principal opening, the others having closed. Before the cavity had entirely healed, a few drops of pus found exit at the nipple. Meantime, the left breast, which had continued more or less hard on the axillary side, suppurated, and an opening was made on March 30, which discharged about four ounces of pus, and a few sloughy shreds. This breast had continued to secrete milk. It was daily rubbed and dressed with the sponges, like the other. There was little pain or soreness near the seat of the abscess. On the sternal side, however, inferiorly, and at the superior edge of the gland, there were two points where the patient complained of considerable tenderness on pressure. There was also some induration at these points, which felt to the touch like the distended vessels of the cord in variocele. After rubbing them for some minutes in the direction of the nipple, a considerable quantity of thick milk was first discharged through the natural orifice, followed by a few drops of pus. Evidently, the matter had formed within and at the extremities of the milk tubes, and had been there imprisoned. The same phenomenon presented itself for several successive days, the quantity of pus gradually diminishing and that of the milk, from this portion of the organ, increasing. At length, all purulent discharge ceased, and with it all induration and soreness.

Finally, on April 8, all the wounds were entirely closed; all induration had disappeared, and the patient had entirely recovered her health.—There was milk, apparently of good quality, in both breasts—the right secreting a little, the left freely. An attempt was made to induce the child to nurse, but having been kept from the breast so long, it refused to lay hold of the nipple. After an interval of three months, the breasts continued to secrete a little. They were entirely healthy, the cicatrices scarcely noticeable.

Although this case was of longer duration than either of the others which I have related, I consider it as furnishing fully as valuable testimony to the merits of the treatment as any of them. Every application of the sponges was followed by marked improvement, while that and the daily frictions brought about a restoration of the suspended secretion. A very interesting point is the evidence furnished that pus may form at the extremities of the milk tubes, and be discharged through the nipple, requiring no other outlet.

I suspect that, in this case, the sloughs were formed at a very early period. The comparative absence of tenderness, combined with such extensive induration, leads me to entertain this opinion. The duration of the case was probably doubled by their occurrence.

*Case* 4.—Mrs. C. confined with her fourth child June 12, 1857. This patient had been in very feeble condition for a long time. Soon after the birth of the last child, about three years since, I had detected tubercles in her lungs, and during the whole of this gestation she had copious and persistent hæmoptysis. She got along pretty well, however, during her labor, and afterward for about a fortnight, when she had one or two poroxysms apparently of intermittent fever, which was relieved by quinine. A day or two after, the right breast became painful and inflamed, and a small abscess formed near the periphery of the gland, toward the sternum. This was opened, discharged about a drachm and a half of pus, and healed without difficulty. Two days later there was renewed pain and tumefaction, chiefly on the opposite side of the breast; the whole breast became hard, then œdematous, and I had no doubt of the existence of matter, though it did not point. About the same time the saphena vein of the right leg became inflamed, for a space of about four inches, near the knee. The patient was extremely averse to the lancet, and I, therefore, seeing that there was no certainty of reaching the matter without an extensive cut, contented myself with poulticing for several days, treating, meanwhile, the phlebitis, which got better. At length, detecting unequivocal fluctuation on the outer side of the breast, I made an opening with a tenotomy knife, running it in nearly an inch and a half, but nothing followed except blood and a little turbid-looking serum. The cut bled a good deal during the day, and weakened the patient considerably. Up to this time I had been unwilling to use the sponges on account of the difficulty in the lungs, fearing, lest the wet condition in which the patient's chest must necessarily be, might light up the old trouble, which seemed now quiescent. But having examined her chest carefully, and found much less evidence of extensive disease than I had anticipated, I decided to take the risk, and on July 13, the day following the second incision, applied them so as to compress the whole breast. In twenty-four hours the œdema had almost entirely disappeared; in two days more, the milk, which had been entirely stopped, began to flow. I continued to apply the sponges daily for a week, very much to the comfort and satisfaction of the patient, who said that nothing had given her so much relief. On the 20th, I applied them for the last time, the patient going into the country next day, with the breast everywhere nearly as soft as the other, and the milk flowing very freely. I should add that, on this day, I lanced a small superficial abscess on the axillary edge of the breast, which had been observed since the second day of the application of the sponges, letting out only half a drachm of matter. There was no bad effect whatever upon the pulmonary disease. If there was any collection of matter in the substance of the breast, it had become absorbed.

In this case the sponges used were not compressed, but were applied after being wrung out of water, and subsequently saturated. They are not quite so conveniently applied as when compressed, and the pressure cannot be so equally made; but they will probably answer pretty well under some circumstances.

The secret of the efficacy of the sponges consists, it seems to me, in three things :—1. They enable us to apply uniform, and at the same time gentle, pressure. 2. They have all the emolient effect of a poultice. 3. They absorb the matter or milk discharged, and promote its flow by capillary attraction.

It is proper to add that I have occasionally, in all these cases, used tents for a day or two to keep open wounds which showed a tendency to heal before it was desirable. A bit of sponge answers admirably for this purpose.

There can be no question, I think, of the superiority of the mode of treating breast abscesses, which is here described, over the old methods by the stimulating injection, the perforating seton, and the large and deep incisions. The profession must decide also whether it is not also better than that of M. Chassaignac. Let us compare the advantages and disadvantages of the two methods :—

1. The method by occlusion produces obliteration of the cavity in one or two days in the superficial abscesses. Our method does the same. Here we are equal.

2. The daily injections of tepid water, and the applications of the cupping glass are attended, according to M. C.'s own statement, with very severe pain, rendering the administration of chloroform almost indispensable. Our method is attended with no pain whatever, so far as I have seen; but, on the contrary, with an immediate sense of comfort and relief. (I attribute the pain which existed, for a short time, in some of Dr. Johnson's cases, to his having employed cold instead of tepid water in moistening the sponges.)

3. The method of M. C. requires the use of apparatus not always at hand; the irrigator, the hydrocele syringe, or the "*sonde a injection recurrente,*" the cupping instrument, etc. We require nothing but sponges and bandages.

4. The French surgeon is obliged, occasionally, to resort to the perforating seton. With us this has not been required, nor have we thus far been obliged to make even a counter opening.

5. It has never been found necessary with us to confine the arm, though perhaps that would still further accelerate the cure.

6. The average duration of the cases of deep-seated abscesses is considerably reduced by our method.

We have little or no information, in M. Chassaignac's reports, in regard to the secretion of milk. In all the cases above related it was preserved or restored.—*New York Journal of Medicine.*

